# Reversible acute heart failure induced by thyrotoxic cardiomyopathy: A case report

**DOI:** 10.1097/MD.0000000000038305

**Published:** 2024-05-24

**Authors:** Rui Shi, Wenyi Meng, Jinzhu Yin, Wen Xue, Yue Deng

**Affiliations:** a Department of Cardiovascular Medicine, Changchun University of Chinese Medicine, Changchun, China; b Department of Emergency Medicine, Affiliated Hospital, Changchun University of Chinese Medicine, Changchun, China; c TCM Cardiovascular Clinical Medicine Research Center of Jilin Province, Changchun, China.

**Keywords:** atrial fibrillation, case report, preexcitation syndrome, thyrotoxic cardiomyopathy, thyrotoxicosis

## Abstract

**Rationale:**

Thyrotoxic cardiomyopathy is a rare but severe complication of thyrotoxicosis, leading to episodes of acute heart failure. This case report highlights a rare presentation of thyrotoxic cardiomyopathy with low-output heart failure, emphasizing the importance of early diagnosis and comprehensive management. The report aims to increase awareness among clinicians about the potential reversibility of this condition and the effective strategies for managing such complex cases.

**Patient concerns:**

This patient presented with dyspnea and chest constriction, without any antecedent predisposing factors. Subsequently, the patient abruptly manifested symptoms indicative of acute heart failure during outpatient consultation. Electrocardiography revealed rapid atrial fibrillation with type A preexcitation syndrome, whereas cardiac ultrasonography demonstrated global cardiac enlargement with a diminished ejection fraction (EF).

**Diagnoses:**

After a comprehensive evaluation, the patient was diagnosed with thyrotoxic cardiomyopathy, acute heart failure, and atrial fibrillation with preexcitation syndrome.

**Interventions:**

Immediate interventions comprised diuretic administration, oxygen therapy, and antiarrhythmic agents, addressing acute heart failure concomitant with preexcitation syndrome. Following a fortnight of comprehensive therapeutic measures, the patient was discharged with a prescription for oral medications, notably methimazole.

**Outcomes:**

Following the intervention, the patient showed significant improvement with the resolution of heart failure symptoms and dyspnea, restoration of sinus rhythm, improved left ventricular ejection fraction (LVEF improved from 36% to 45%), and normalization of thyroid function. These outcomes underscore the efficacy of the intervention strategy and offer a hopeful prognosis for similar cases.

**Lessons:**

Thyrotoxicosis may cause cardiomyopathy in patients with heart failure that manifests as dilated cardiac chambers. Clinicians should carefully screen patients for this reversible condition. Diagnosis requires a comprehensive assessment of various tests, and the therapeutic goal is to restore normal thyroid function.

## 1. Introduction

The thyroid gland, functioning as an endocrine organ, secretes triiodothyronine (T3), thyroxine (T4), and calcitonin (CT).^[1]^ Its secretion governs various physiological processes, including basal metabolic rate, growth and development, and modulation of the autonomic and respiratory systems.^[2]^ Thyrotoxic cardiomyopathy is an infrequent complication of thyrotoxicosis that poses a heightened mortality risk to patients.^[3]^ The initial manifestation of cardiomyopathy has been documented in 6% of cases, with <1% progressing to severe left ventricular dysfunction.^[4]^ Myocardial injury in thyrotoxic cardiomyopathy primarily stems from the hyperstimulatory impact of surplus thyroid hormones, particularly T3.^[5]^

Elevated levels of circulating T3 precipitate heightened cardiac output by augmenting myogenic fiber contractility (output per beat) and heart rate,^[6]^ culminating in an increased myocyte metabolic rate. Furthermore, the heightened output per beat corresponds to an increase in total blood volume. The effect of T3 on vascular smooth muscle induces peripheral vasodilation,^[7]^ reduces systemic vascular resistance, and subsequently activates the renin-angiotensin system, thereby retaining body fluid and salt.^[8,9]^ Additionally, T3 fosters erythropoiesis, contributing to an overall net augmentation in total blood volume and per-pulse output.^[10]^ Collectively, these mechanisms result in a high-output cardiac state that may precipitate manifestations associated with heart failure.

## 2. Case report

### 2.1. Patient information

A 36-year-old female presented with dyspnea and chest constriction that had persisted for 20 days without any preceding predisposing factors. Progressive exacerbation of dyspnea and chest constriction was accompanied by nocturnal bouts of dyspnea and limb weakness lasting for 20 days. The patient denied experiencing fever or palpitations. The patient had no history of hypertension or cardiac ailments. No relevant treatment was undertaken before admission, and there was no record of prior surgeries or medications. The patient lacked pertinent personal or familial medical history. The patient exhibited acutely distressed countenance, lip cyanosis, marked lower extremity edema, blood pressure of 122/68 mm Hg, respiratory rate of 34 breaths/minutes, scattered moist rales in the bilateral lower lung fields, tachycardia at 188 beats/min, irregular rhythm, and discernible gallop rhythm upon cardiac auscultation.

**Figure 1.**
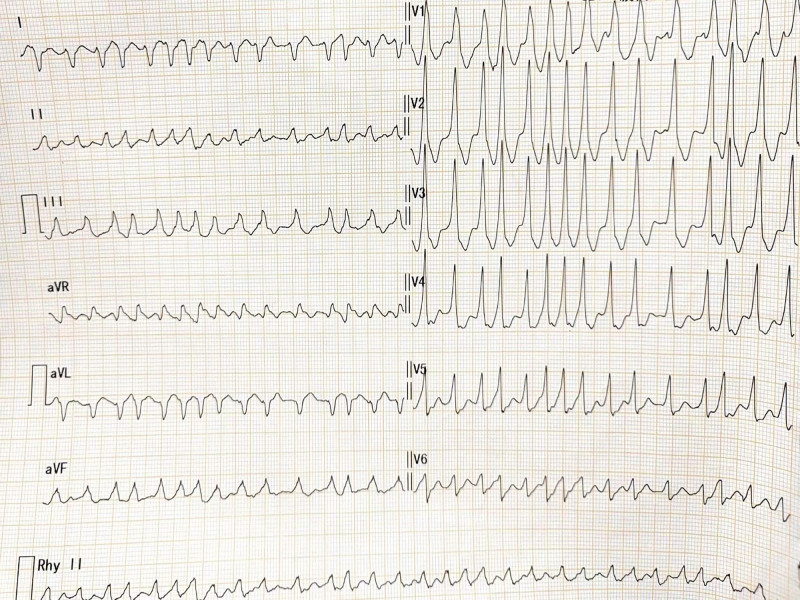
Electrocardiogram at first visit. Rapid-type atrial fibrillation, type A preexcitation syndrome, ST-segment downshift, T-wave inversion in some leads.

**Figure 2.**
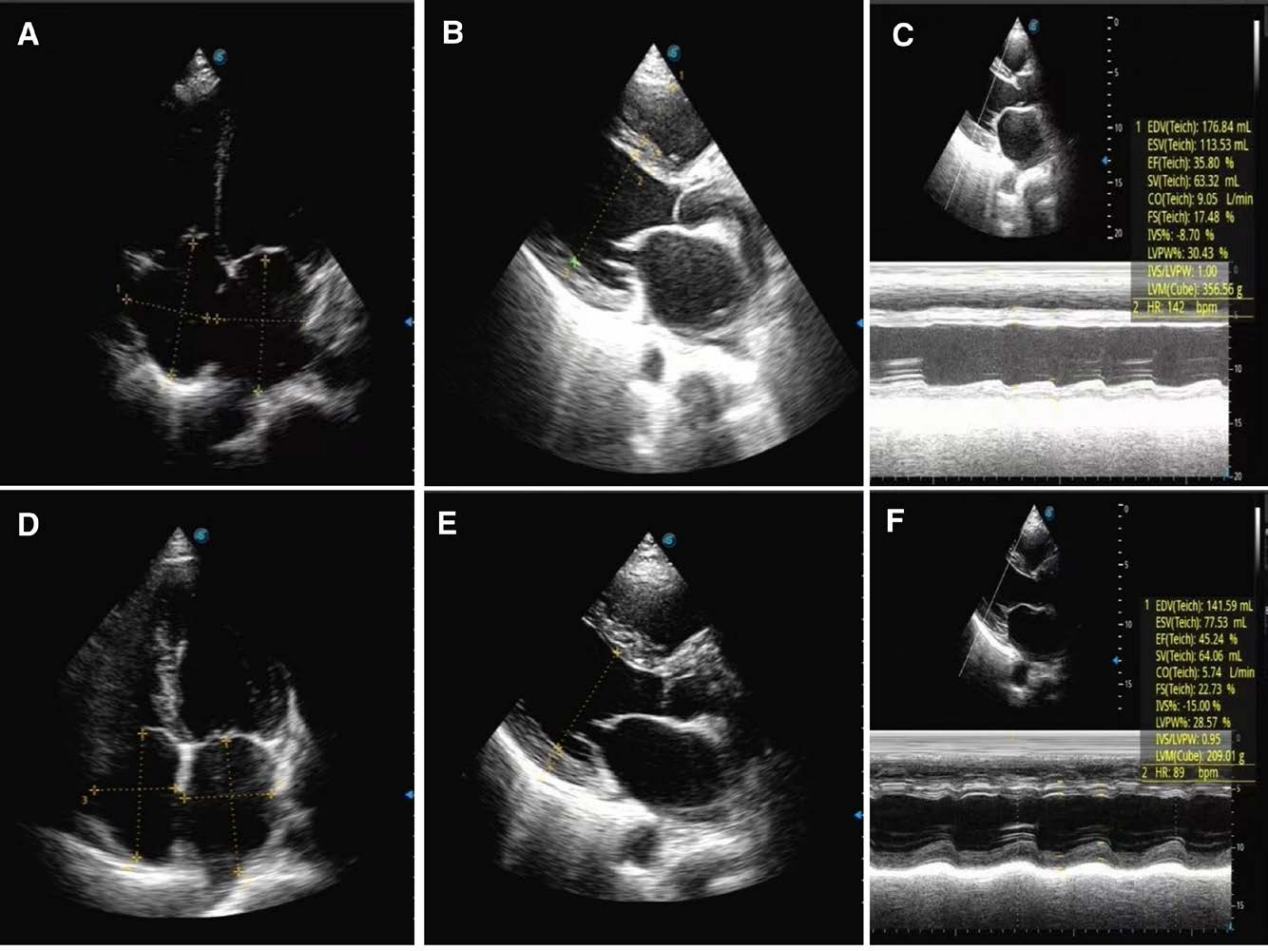
Echocardiograms from the first visit to the last follow-up. (A) Apical 4-chamber view at first visit; (B) Left ventricular long-axis view at first visit; (C) Indices of echocardiogram at the first visit. (D) Apical 4-chamber view at 1-mo follow-up. (E) Left ventricular long-axis view at 1-mo follow-up. (F) Indices of echocardiogram at 1-mo follow-up.

**Figure 3.**
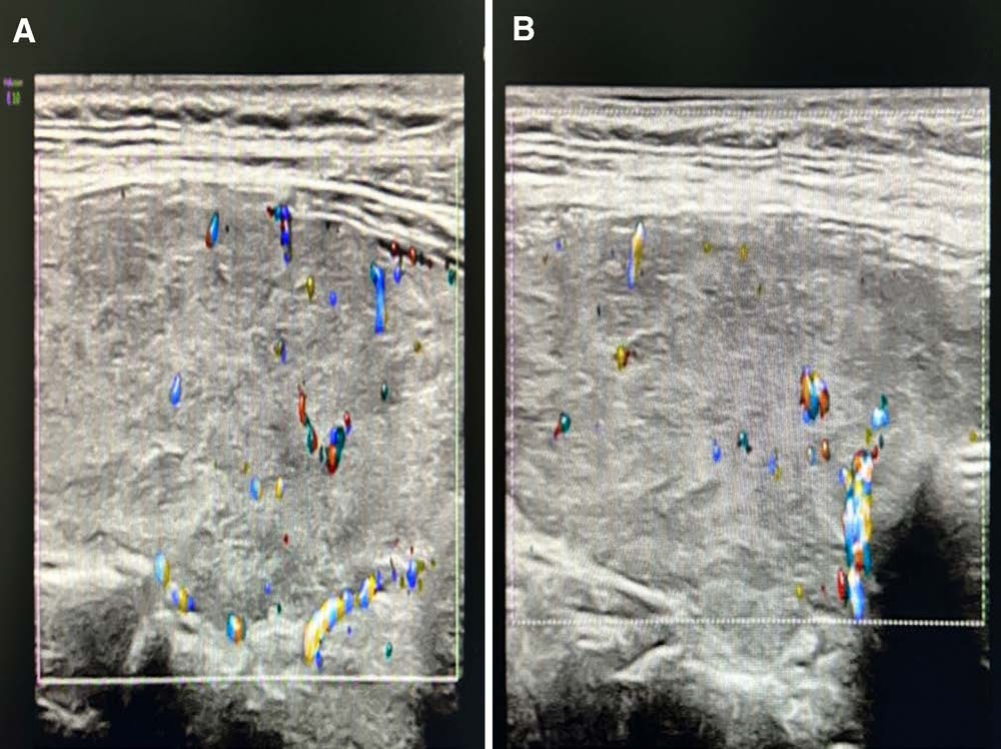
Comparison of thyroid ultrasound between the first visit and the last follow-up. (A) Thyroid ultrasound findings at the first visit. (B) Thyroid ultrasound findings at follow-up.

Outpatient laboratory examinations: erythrocyte 5.12*10^12^/L (normal range 3.8–5.1^12^/L). Liver function: total bilirubin 43.6 µmol/L (normal range 3.4–20.5 µmol/L), direct bilirubin 20.5 µmol/L (normal range 0.5–8.6 µmol/L), indirect bilirubin 23.1 µmol/L (normal range 1.7–17 µmol/L). N-terminal pro-b-type natriuretic peptide (NT-proBNP) was 4021 pg/mL (normal range 0–450 pg/mL). Cardiac enzyme and troponin levels were within normal ranges, ruling out acute myocarditis and acute myocardial infarction. Outpatient electrocardiography (Fig. 1) revealed a rapid atrial fibrillation rhythm with type A preexcitation syndrome. Outpatient echocardiography (Fig. 2A–C). The left ventricular end-systolic volume was 113 mL, end-diastolic volume was 176 mL, the right ventricular anterior–posterior diameter was 31 mm, left atrial index was (LAVI) 19.9 mL/m^2^, left ventricular ejection fraction (LVEF) was 36%. M: Diffuse hypokinesia of ventricular wall motion and mitral regurgitation over an area of 12 cm^2^. This suggests an enlargement of the entire heart. Severe mitral valve regurgitation and impairment of left ventricular systolic function impairment outpatient chest CT showed calcified spots in the upper lobe of the right lung and an enlarged heart. Thyroid ultrasound (Fig. 3A) revealed thyroid parenchyma with uneven echogenicity and increased blood flow.

### 2.2. Initial diagnosis and treatment

The patient experienced an abrupt escalation in dyspnea severity during an outpatient consultation and was initially diagnosed with dilated cardiomyopathy (DCM), atrial fibrillation with preexcitation syndrome, and acute heart failure, as ascertained through an electrocardiogram, cardiac ultrasound, and an anomalous NT-proBNP finding.

Immediate resuscitation measures were initiated in the emergency department. Multifunctional monitoring indicated that the patient’s blood pressure fluctuated at approximately 134/60 mm Hg, respiratory rate was 38 breaths/minute, oxygen saturation was maintained at 93%, and heart rate was 190 beats/minute. Numerous dry and wet rales were auscultated in both lung fields, and a furosemide injection 40 mg IV was administered immediately to improve the pulmonary edema. Oxygen support was provided because the patient had rapid atrial fibrillation with preexcitation syndrome, and digitalis glycoside drugs were not administered to prevent possible ventricular fibrillation. After the administration of esmolol hydrochloride, the patient’s heart rate did not decrease significantly, and the patient rapidly developed acute heart failure decompensation. The patient presented in a forced sitting position with severe dyspnea, oxygen level of 88%, respiratory rate of 40 breaths/minute, heart rate of 156 beats/minute, and blood pressure of 126/69 mm Hg. Following confirmation of the absence of a history of bronchial asthma, 5 mg morphine was intramuscularly administered and 5 mg dexamethasone sodium phosphate was intravenously administered. Administered intravenous dihydroxypropyltheophylline 0.5 g of solution was administered intravenously to the patient to relieve airway spasms, and subsequently, recombinant human brain natriuretic peptide was injected to ameliorate heart failure. Following a loading dose of 1.5 µg/kg administered over 5 minutes, continuous infusion at a rate of 0.01 µg/kg/minute was initiated. After 30 minutes of resuscitation therapy, the patient’s heart rate remained above 140 beats/minute and rapid atrial fibrillation with preexcitation syndrome persisted. Consequently, amiodarone hydrochloride injection was initiated. Initially, 150 mg was administered intravenously for 10 minutes, followed by a subsequent infusion of 360 mg at a rate of 1 mg/minute. Following approximately 2-hours of resuscitation therapy, the patient acute heart failure was successfully corrected. Monitoring revealed an oxygen saturation of 96%, respiratory rate of 25 breaths/minute, heart rate of 126 beats/minute, and blood pressure of 132/74 mm Hg. The patient electrocardiographic rhythm persisted with atrial fibrillation; however, there were no signs of preexcitation syndrome. An improvement in dyspnea was noted, and low-molecular-weight heparin was administered to prevent thrombus formation. Calcium injections (5000 IU) were administered subcutaneously twice daily. Concurrently, alterations in the coagulation function were observed. Subsequently, propranolol hydrochloride was administered orally at a dose of 20 mg 3 times daily, accompanied by a minimal dosage of furosemide tablets (20 mg) and daily oral intake of spironolactone (20 mg). The patient received 20 mg methimazole tablets orally twice daily. Following a 2-week course of treatment, the patient exhibited robust recovery, marked by substantial amelioration of symptoms associated with heart failure, notably dyspnea. The patient was discharged with a prescription for oral medication.

### 2.3. Final diagnosis

Laboratory examinations on the second day: thyroid-stimulating hormone < 0.005 µIU/mL (normal range 0.27–4.2 µIU/mL), serum free T3 30.02 pmol/L (normal range 3.1–6.8 pmol/L), serum free T4 96.22 pmol/L (normal range 12–22 pmol/L), and antithyroid peroxidase antibody 270.90 IU/mL (normal range 0–34 IU/mL), NT-proBNP 3381 pg/mL (normal range 0–450 pg/mL). Coronary angiography did not reveal any stenosis of the coronary lumen, ruling out coronary artery disease. Upon evaluating the patient’s symptoms, conducting a thorough physical examination, considering the lack of previous medical history, and analyzing laboratory and imaging results such as thyroid function, the conclusive diagnosis is reached through a process of elimination encompassing the following conditions: thyrotoxic cardiomyopathy, acute heart failure, and atrial fibrillation with preexcitation syndrome.

### 2.4. Outcome and follow-up

**Figure 4.**
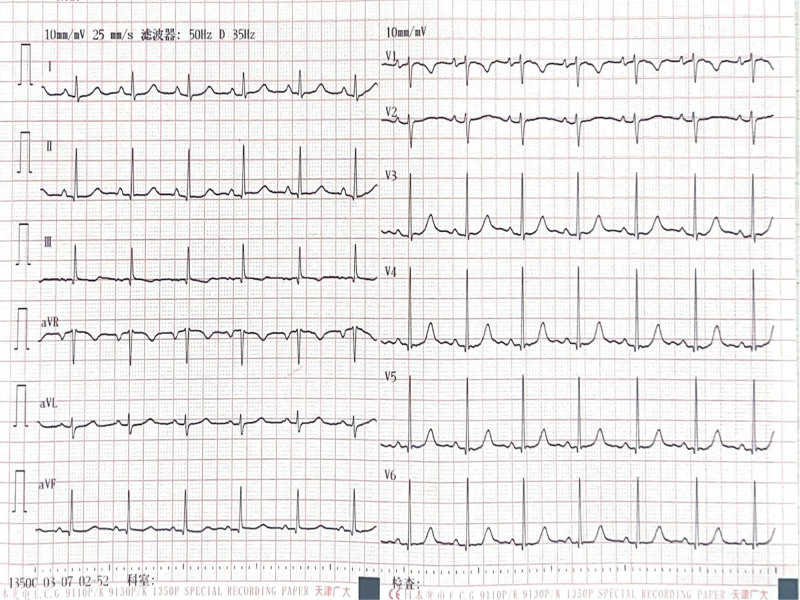
Electrocardiogram at the last follow-up. Sinus rhythm, normal ECG.

**Figure 5.**
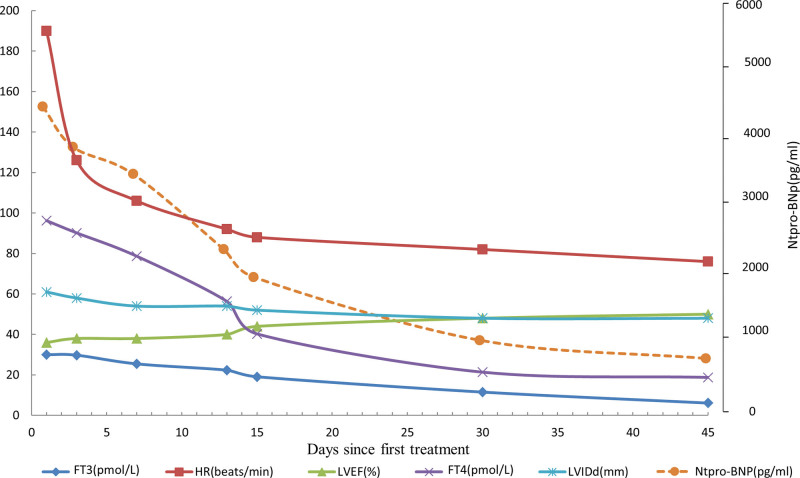
Changes in indicators during treatment and at follow-up. FT3 = free triiodothyronine, FT4: free thyroxine, HR = heart rate, LVEF = left ventricular ejection fraction, LVIDd = left ventricular internal dimension in diastole, Ntpro-BNP = N-terminal pro-b-type natriuretic peptide.

During the 1-month post-discharge follow-up period, the patient had no symptoms of heart failure or dyspnea. Physical examination revealed an absence of overt positive signs. Electrocardiography (Fig. 4) indicated the restoration of normal sinus rhythm with effective heart rate control. A review of the thyroid function revealed a return to normal levels. The NT-proBNP was 321 pg/mL (reference range 0–450 pg/mL); and notable alterations in the patient indices were evident compared with the initial admission, as illustrated in Figure 5. Evaluation of the echocardiograms (Fig. 2D–F) revealed significant changes compared with the initial visit. The left ventricular end-systolic volume was 78 mL, end-diastolic volume was 141 mL, the right ventricular anterior–posterior diameter was 27 mm, and the left atrial index (LAVI) was 14.2 mL/m^2^, LVEF was 45%. The mitral regurgitation area had contracted to 1.7 cm^2^. Subsequent thyroid ultrasound (Fig. 3B) revealed heterogeneous parenchymal echogenicity in the thyroid gland, and the blood flow signal had decreased compared to the prior examination.

## 3. Discussion

This substantiation indicates that euthyroid conditions constitute an optimal milieu for cardiovascular homeostasis.^[11]^ The nexus between thyrotoxicosis and the incidence of cardiovascular pathology has been elucidated, and high-output manifestations of thyrotoxicosis include cardiomegaly, atrial fibrillation, high-output cardiac failure, hypertrophic cardiomyopathy, angina pectoris without substantiation of coronary artery disease, and sudden demise.^[12,13]^ The high-output constellation in thyrotoxicosis is precipitated by the immediate potentiation of the heart rate and contractility by thyroid hormones, which secondarily augment blood volume and engender peripheral vasodilation.^[14]^ Notwithstanding the antecedent of increased cardiac performance, high-output cardiac failure persists, evolves, and exacerbates.

DCM is characterized by cardiac enlargement and myocardial debilitation, which culminate in diminished cardiac pumping efficacy. This ailment can be genetic or induced by various factors including infection, toxin exposure, and drug responses.^[15]^ The current instance represents the occurrence of thyrotoxic cardiomyopathy, an uncommon sequela of thyrotoxicosis, in approximately 1% of patients with thyrotoxicosis and thyrotoxic cardiomyopathy (TCM), which may precipitate profound left ventricular dysfunction and culminate in cardiogenic shock, carrying a substantial mortality risk.^[16]^ Cardiac myopathy typically manifests as a “high-output” form of cardiac insufficiency, wherein cardiac output may surge by 50% to 300% due to a multifaceted interplay of basal heart rate, contractility, ejection fraction, augmented systolic volume, and reduced systemic vascular resistance, whereas instances of low-output cardiac failure remain scarce.^[17]^

Owing to the patient’s precarious status, an expeditious diagnosis was imperative and necessitated prompt supportive measures and resuscitation. In this case, thyrotoxicosis management culminated in the stepwise retrieval of left ventricular function. Recognition of this potential etiopathogenesis and representation of thyrotoxicosis can facilitate the identification of this distinct cohort of patients with reversible DCM. However, the prognosis of this variant of cardiomyopathy secondary to thyrotoxicosis requires stringent follow-up and surveillance. Certain investigations have documented that cardiomyopathy in patients with thyrotoxicosis can still cause irreversible sequelae despite efficacious intervention.^[18]^

The patient manifested concomitant atrial fibrillation and type A preexcitation syndrome associated with heart failure. Thyrotoxicosis is incontrovertibly linked to a substantial risk of atrial fibrillation. The thyroid regulates cardiac structural, functional, and electrical remodeling, as shown in experimental studies at the cellular level and in animal models of thyroid disease.^[19,20]^ The thyroid affects atrial physiology through both endocrine and paracrine mechanisms, rendering the atrial myocardium more sensitive, promoting atrial fibrosis, and increasing the propensity for atrial fibrillation.^[21,22]^

Thyrotoxicosis is a reversible precipitant of atrial fibrillation, with evidence showing that approximately 2-thirds of individuals with thyrotoxicosis experience a spontaneous return to sinus rhythm following the restoration of thyroid hormone levels.^[23]^ In our case, the patient was diagnosed with thyrotoxic cardiomyopathy and was subsequently admitted to the hospital because of an episode of acute heart failure. The primary etiology was identified as DCM during the initial assessment. Although beta-blockers are the preferred pharmacological intervention for heart rate reduction in patients with hyperthyroidism, the heart rate remains unresponsive to beta-blockers administered over a brief interval. Consequently, amiodarone hydrochloride injection was used to achieve partial heart rate control in the absence of thyroid function test results. Although hyperthyroidism stands out as a commonly diagnosed adverse effect in patients taking amiodarone,^[24]^ the transient administration of amiodarone could potentially contribute to recovery from acute heart failure in this patient. This is attributed to the elevated iodine content, which results in diminished iodine uptake by the thyroid gland. Concurrently, this impedes the synthesis of thyroid hormones, culminating in reduced hyperthyroidism. Subsequent therapeutic interventions include the administration of methimazole to treat hyperthyroidism.

This category of medications primarily inhibits the synthesis of thyroid hormones by predominantly targeting thyroid peroxidase in hyperactive thyroid tissues.^[25]^ This mechanism diminishes T3 and T4 levels, thereby ameliorating the unfavorable prognosis of thyrotoxicosis and associated heart failure. Anticoagulant agents were administered during the course of treatment, and heightened susceptibility to thrombotic occurrences in patients with atrial fibrillation and hyperthyroidism has been demonstrated to be unrelated to CHADS2-VASc scoring.^[26]^

Nevertheless, the management of hyperthyroidism and anticoagulation in patients with atrial fibrillation remains controversial, owing to the impact of thyroid dysfunction on blood flow regulation, which consequently influences the effectiveness of anticoagulation in the affected population. Additionally, the use of anticoagulants may introduce uncertainty regarding the risk of bleeding. Consequently, after heparin therapy, alterations in the patient’s coagulation function were meticulously monitored.

## 4. Conclusion

Herein, we present a case of DCM concomitant with atrial fibrillation and type A preexcitation syndrome, which culminated in an acute episode of heart failure. The patient’s life was safeguarded through emergency intervention, and a comprehensive diagnostic approach was used to elucidate the etiology of thyrotoxic cardiomyopathy. The continuous administration of medications to control hyperthyroidism gradually restored cardiac function and electrocardiographic manifestations to normal levels. Severe hyperthyroidism can precipitate the onset of dilated thyrotoxic cardiomyopathy, a rare yet perilous cardiac condition that can be effectively mitigated within a brief period through timely and appropriate interventions. Hence, clinicians should remain vigilant regarding the potential presence of this uncommon ailment in patients presenting with recently diagnosed heart failure, particularly when accompanied by unexplained tachyarrhythmia or indications of DCM. Furthermore, our case highlights a potentially favorable outcome associated with the short-term use of amiodarone in managing thyroid storms.

This case report describes a single patient experience, which may not fully represent the broader population affected by thyrotoxic cardiomyopathy. The findings are based on observational and retrospective data, which limits the ability to establish causation. Additionally, the management approach detailed here may not be applicable in all clinical settings due to varying resources and expertise available.

## Author contributions

**Conceptualization:** Rui Shi, Wenyi Meng, Yue Deng.

**Data curation:** Rui Shi, Jinzhu Yin.

**Formal analysis:** Rui Shi, Jinzhu Yin, Wen Xue.

**Supervision:** Yue Deng.

**Writing – original draft:** Rui Shi, Wenyi Meng, Yue Deng.

**Writing – review & editing:** Rui Shi, Wen Xue, Yue Deng.
